# Evaluation of supply-side initiatives to improve access to coronary bypass surgery

**DOI:** 10.1186/1472-6963-12-311

**Published:** 2012-09-11

**Authors:** Boris G Sobolev, Guy Fradet, Lisa Kuramoto, Rita Sobolyeva, Basia Rogula, Adrian R Levy

**Affiliations:** 1 School of Population and Public Health, The University of British Columbia, Vancouver, BC, Canada; 2 Department of Surgery, The University of British Columbia, Vancouver, BC, Canada; 3 Centre for Clinical Epidemiology and Evaluation, Vancouver Coastal Health Research Institute, Vancouver, BC, Canada; 4, Utilitas Consulting, Vancouver, BC, Canada; 5 Community Health & Epidemiology, Dalhousie University, Halifax, NS, Canada

**Keywords:** Access to care, CABG, Surgical wait lists, Provincial registry, Health policy

## Abstract

**Background:**

Guided by the evidence that delaying coronary revascularization may lead to symptom worsening and poorer clinical outcomes, expansion in cardiac surgery capacity has been recommended in Canada. Provincial governments started providing one-time and recurring increases in budgets for additional open heart surgeries to reduce waiting times. We sought to determine whether the year of decision to proceed with non-emergency coronary bypass surgery had an effect on time to surgery.

**Methods:**

Using records from a population-based registry, we studied times between decision to operate and the procedure itself. We estimated changes in the length of time that patients had to wait for non-emergency operation over 14 calendar periods that included several years when supplementary funding was available. We studied waiting times separately for patients who access surgery through a wait list and through direct admission.

**Results:**

During two periods when supplementary funding was available, 1998–1999 and 2004–2005, the weekly rate of undergoing surgery from a wait list was, respectively, 50% and 90% higher than in 1996–1997, the period with the longest waiting times. We also observed a reduction in the difference between 90th and 50th percentiles of the waiting-time distributions. Forty percent of patients in the 1998, 1999, 2004 and 2005 cohorts (years when supplementary funding was provided) underwent surgery within 16 to 20 weeks following the median waiting time, while it took between 27 and 37 weeks for the cohorts registered in the years when supplementary funding was not available. Times between decision and surgery were shorter for direct admissions than for wait-listed patients. Among patients who were directly admitted to hospital, time between decision and surgery was longest in 1992–1993 and then has been steadily decreasing through the late nineties. The rate of surgery among these patients was the highest in 1998–1999, and has not changed afterwards, even for years when supplementary funding was provided.

**Conclusions:**

Waiting times for non-emergency coronary bypass surgery shortened after supplementary funding was granted to increase volume of cardiac surgical care in a health system with publicly-funded universal coverage for the procedure. The effect of the supplementary funding was not uniform for patients that access the services through wait lists and through direct admission.

## Background

In Canada, provincial health insurance plans provide universal, single-payer coverage for surgical coronary revascularization, a procedure indicated for the treatment of coronary artery disease [1]. Responsible for the delivery of care, regional Health Authorities budget a fixed number of open heart surgeries on an annual basis using population-based rates of the disease [2]. As argued elsewhere, those who make allocation decisions have no tools to predict the volume of demand at each hospital and at each point during the calendar period [3]. When demand exceeds funded capacity, cardiac centers across Canada use wait lists to manage access to the procedure. As a result, operations for patients with less severe coronary artery disease may be delayed when a surgical service experiences an extended demand for more urgent procedures [4-10]. Guided by the evidence that delaying the operation may lead to symptom worsening and poorer clinical outcomes, expansion in cardiac surgery capacity has been recommended in Canada. Federal and provincial governments started providing one-time and recurring increases in budgets for additional open heart surgeries to reduce the number of patients waiting for coronary artery bypass grafting (CABG) and their waiting times.

In a previous study of access to non-emergency CABG in British Columbia, Canada, between 1991 and 2000, we found that waiting times for the procedure shortened after 1998 when annual supplementary funding was granted to tertiary care hospitals that had been providing cardiac surgical care to adult residents of the province [8]. Between 1995–1996 and 1999–2000, there was a 12% increase in the total number of CABG operations and a decline in median waiting time from 15 to 10 weeks, although the change in waiting times was different across urgency groups. In addition, between 1995–1996 and 1999–2000 there was a decrease from 54% to 41% in the proportion of patients undergoing the procedure through wait lists. Considering that cardiac surgeons in British Columbia have discretion for direct admission of their patients on the basis of the estimated urgency of treatment, place of residence and other factors, this might indicate that supplementary funding had been used to provide more treatments without delay. One plausible explanation for these results was that the hospitals had capacity to increase the number of operations and thereby reduce wait times [10]. However, a limitation of the previous study was the short period for analysis of the effects of supplementary funding (two years). In addition, data were not available for the total amount of time between cardiac catheterization and surgical revascularization. As such, in the previous study we did not estimate the effect of the supplementary funding on waiting times in full.

Since our previous analysis, another $2 million of additional funding from the provincial Ministry of Health was directed in each of 2003 and 2004 toward open heart surgery to increase the volume. However, the effect of this increase in funding on wait-list sizes and waiting times for CABG remains unknown. The longer time frame after the original increase in annual budgets for CABG since 1998 and the additional funding in 2003 and 2004 makes it feasible to generate more precise assessment of this supply-side initiative to improve access to care. The effect of the additional funding has not been contrasted for two different pathways of accessing surgical coronary revascularization for non-emergency patients as well. Access to non-emergency CABG could be provided either through a wait list or through direct admission to hospital, and it remains unclear whether the effect of the supplementary funding was uniform for the wait-listed and directly-admitted patients. However this information is important for deciding on policy to reduce wait lists by adding extra funding.

To evaluate the effect of providing the additional funding to tertiary care hospitals on access to non-emergency surgical revascularization within a single publicly funded health system, we estimated temporal changes in the length of time between decision to proceed with surgery and performed CABG. We used all relevant records from the provincial population-based registry of patients with angiographically-proven coronary artery disease identified as needing bypass surgery on a non-emergency basis. To adjust for changes in time between cardiac catheterization and decision to operate, we used the most recent catheterization date from hospital discharge reports assuming that the results of this procedure (coronary angiography or intervention) were most likely linked to the decision to operate. Primary comparisons have been done across synthetic cohorts of patients defined by the calendar period of the decision to proceed with surgery. The temporal changes in treatment delays have been estimated separately for patients who access surgery by registration on a wait list and among patients who access surgery by direct admission.

## Methods

### Data sources

Data were obtained from the British Columbia Cardiac Registries (BCCR) to identify the study participants and their characteristics. This population-based patient registry contains demographic, clinical and treatment data, along with the dates of booking request for operating room time and procedures for all adult patients undergoing CABG in any of the four cardiac centers in the province [8]. To identify cardiac catheterization dates, hospital admission and discharge dates, and coexisting medical conditions, we used each patient’s provincial health number to deterministically link BCCR records to the Canadian Institute for Health Information (CIHI) Discharge Abstract Database (DAD) [11].

### Patients

Using records from the registry, we studied two groups of patients: (1) those who were registered on a wait list for first-time isolated CABG surgery; and (2) those who underwent the procedure by direct admission to hospital on a non-emergency basis. Patients who accessed surgery through a wait list were registered by the surgeon’s office on the wait list after an outpatient consultation with a cardiac surgeon. In contrast, patients who accessed surgery through direct admission were admitted to a hospital’s cardiac ward directly from the catheterization laboratory or after an outpatient consultation with a cardiac surgeon if the patient had disabling symptoms or high-risk anatomy of the coronary lesion(s). Patients in both groups were classified as urgent, semiurgent, or nonurgent based on the patient’s need for treatment, as defined elsewhere [8].

The inception cohort of wait-listed patients had a total of 14,049 records of registration for CABG from January 1, 1991 through December 31, 2005. We excluded 567 records of patients for various reasons: procedure at registration was not isolated CABG (312), procedure at registration or at surgery was not first-time CABG (62), emergency cases at the time of registration (34), missing operating room reports (4), removed on the registration date (101), registration was on a weekend and admission was the day after (14), or the patient had multiple episodes (40). We also excluded 1,452 records of patients who were registered in 1991 (797) or did not have a catheterization date (655).

The inception cohort of direct admissions had a total of 16,014 records of CABG surgery from January 1, 1991 through December 31, 2005. We excluded 1,282 records of patients for various reasons: procedure at surgery was not isolated CABG (211), procedure at surgery was not first-time CABG (54), emergency case at the time of surgery (861), or the patient had multiple episodes (156). We also excluded 1,914 records of patients who had surgery in 1991 (1,031), did not have a catheterization date (838), did not have an admission date (38), or had a decision to operate in 1991 (7).

The final study cohort had a total of 12,030 wait-listed patients who had a decision to undergo first-time isolated CABG surgery and 12,818 direct admissions who underwent first-time isolated CABG surgery from January 1, 1992 through December 31, 2005. Among the wait-listed patients 10,339 (85.9%) underwent surgery within 1 year of registration and the remaining were removed from the list without surgery: 104 (0.9%) died, 257 (2.1%) continued to receive medical treatment, 231 (1.9%) declined surgery, 86 (0.7%) were transferred to another surgeon or hospital, 321 (2.7%) were removed for other reasons, and 692 (5.8%) remained on the list after 52 weeks or at the end of the study period.

### Primary study variable

The primary study variable was calendar period of decision to operate classified into 7 two-year periods: 1992–93, 1994–95, 1996–97, 1998–99, 2000–01, 2002–03, 2004–05. Calendar periods are often used as a proxy for changes in the availability of hospital resources, such as, surgical staffing, operating room time, special equipment, and beds, in studies that attempted to explain variations in the patient’s waiting time for surgery [12,13]. Because the budget for open heart surgeries is determined annually, calendar period provides an appropriate indicator for changes in the funded procedures.

### Outcome

The outcome was time between decision to operate and surgical revascularization. This time was measured in calendar weeks for wait-listed patients and in days for direct admissions. We used the date of the registration on a wait list as a proxy for the decision to operate for wait-listed patients and the date of catheterization or the date of admission to hospital, whichever was most recent, as a proxy for the date of decision to operate for directly-admitted patients. This latter rule reflects variation in care paths of the patients: following angiography the patient may be admitted for in-hospital consultation with a cardiac surgeon, or patients who live far away are admitted for angiography, stay in hospital to undergo tests, and are booked for surgery or discharged with planned re-admission.

### Potential confounders

The existing literature suggests that elderly patients are more likely to undergo revascularization as an urgent procedure [14], that smaller diameter of the coronary vessels may account for the higher risk of adverse cardiovascular events among women [15], that co-existing conditions may delay open heart surgery [16], that post-operative survival depends on institutional constraints and individual care providers [17], and that changes in practice may reduce the waiting time until surgery [8]. To identify comorbidities at the time of decision to operate, we used diagnoses reported in the DAD within one year prior to decision. The reference category was defined as no coexisting conditions. The first comparison category was defined as patients with any of the following conditions at presentation: congestive heart failure, diabetes mellitus, chronic obstructive pulmonary disease, cancer, or rheumatoid arthritis [4]. The second comparison category was defined as patients presenting with other coexisting chronic conditions, as defined elsewhere [18].

In addition, we used time between cardiac catheterization and decision to operate as a covariate that might reflect changes in practice over time. The time between catheterization and decision measured in calendar weeks. The catheterization dates were obtained from the DAD and defined as the most recent diagnostic (Canadian Classification of Procedure (CCP) codes 4892–4898, 4996, 4997) or therapeutic (CCP codes 4802, 4803, 4809) catheterization performed within one year preceding and including the date of booking for wait-listed patients or within one year preceding and including the date of surgery for direct admissions. We used the date of most recent catheterization procedures (diagnostic or therapeutic) because the results of this procedure are most likely linked to decision to operate [19]. We used calendar weeks as the unit of time because scheduling of surgical procedures is done on a weekly basis [13].

### Statistical analysis

We used chi-square testing to compare the distributions of patient characteristics across calendar period of decision for wait-listed patients and direct admissions. We estimated percentiles and conditional median times to surgery to characterize the variation in times to surgical revascularization, by calendar period for each type of access. Percentiles of time to surgery were estimated using the product-limit method [13,20]. The conditional median waiting time at a given moment after decision is defined as the period during which one half of the patients who wait for surgery are expected to have it [13]. For wait-listed patients, average weekly surgery rates were calculated as the number of procedures divided by the sum of observed times from decision to surgery or removal from the wait list. For direct admissions, the average daily surgery rates were calculated as the number of procedures divided by the sum of observed times from decision to surgery.

Discrete-time survival regression methods were used to model the relation between the time to surgical revascularization and calendar period of decision for each type of access [13,21]. We restricted the regression analysis to the first 52 weeks after decision for wait-listed patients and to the first 7 days after decision for direct admissions. The calendar period was entered into the regression model as a set of 6 binary indicators. The 1996–1997 group (i.e., the reference group) took a value of 0 for all indicator variables. The exponential of the regression coefficient for an indicator variable for a period gave the odds of surgery in that period relative to the odds of surgery in 1996–1997. In a multivariable model, we adjusted for sex, age at decision, urgency at decision, institution at decision, comorbidities at decision, coronary anatomy at decision, and time between catheterization and decision.

To explore the effect of the supplementary funding on direct admissions and wait-listed patients, we classified all patients using the algorithm developed by Northern New England Cardiovascular Disease Study Group [22]. For each patient, we calculated the prognostic risk of in-hospital death that summarized the effect of clinical and patient characteristics. We then compared the distribution of these risks between direct admissions and wait-listed patients for each calendar period of surgery using chi-square testing.

The Behavioural Research Ethics Board of the University of British Columbia approved the study protocol, Certificate of Approval H06-80651.

## Results

### Patients characteristics

Overall 24,848 patients had a decision for first-time, isolated CABG between 1992 and 2005: 12,030 (48%) were registered on a wait list and 12,818 (52%) were directly admitted to hospital.

For wait-listed patients, the distribution of patient characteristics varied across periods (Table 1). The majority of the wait-listed patients who had a decision for CABG were men (83%). Later periods tended to have older patients (*p*<0.001), fewer urgent cases at decision (*p*<0.001), more patients with major comorbidities at the time of decision (*p*<0.001), and more limited coronary anatomy affected (*p*<0.001). The distribution of cases by institution at decision seemed to increase over periods for hospital 1, but decreased for hospital 2 (*p*<0.001). The majority of wait-listed patients had a decision to operate within a week of catheterization. This majority ranged from about 42% in 1992–1993 and increased to 65% in 2002–2003 (*p*<0.001).

**Table 1:** Characteristics of 12,030 wait-list registered patients, who had decision for coronary artery bypass grafting in British Columbia 1992–2005, by calendar period of decision

	**All periods**		**1992–1993**		**1994–1995**		**1996–1997**		**1998–1999**		**2000–2001**		**2002–2003**												**2004–2005**	
**Characteristic**	**(*n=*12030)**		**(*n=*1726)**		**(*n=*1793)**		**(*n=*1862)**		**(*n=*1610)**		**(*n=*1791)**		**(*n=*1889)**												**(*n=*1359)**	
Age group at decision (years)																
<50	761	(6.3)	140	(8.1)	147	(8.2)	118	(6.3)	99	(6.1)	113	(6.3)	85	(4.5)	59	(4.3)
50–59	2559	(21.3)	362	(21.0)	390	(21.8)	367	(19.7)	337	(20.9)	419	(23.4)	426	(22.6)	258	(19.0)
60–69	4468	(37.1)	703	(40.7)	655	(36.5)	717	(38.5)	551	(34.2)	619	(34.6)	717	(38.0)	506	(37.2)
70–79	3786	(31.5)	500	(29.0)	542	(30.2)	602	(32.3)	567	(35.2)	573	(32.0)	551	(29.2)	451	(33.2)
≥80	456	(3.8)	21	(1.2)	59	(3.3)	58	(3.1)	56	(3.5)	67	(3.7)	110	(5.8)	85	(6.3)
Sex																
Men	9981	(83.0)	1425	(82.6)	1487	(82.9)	1500	(80.6)	1334	(82.9)	1502	(83.9)	1595	(84.4)	1138	(83.7)
Women	2049	(17.0)	301	(17.4)	306	(17.1)	362	(19.4)	276	(17.1)	289	(16.1)	294	(15.6)	221	(16.3)
Urgency at decision∗																
Urgent	739	(6.1)	113	(6.5)	162	(9.0)	177	(9.5)	66	(4.1)	72	(4.0)	87	(4.6)	62	(4.6)
Semiurgent	8769	(72.9)	1331	(77.1)	1240	(69.2)	1295	(69.5)	1107	(68.8)	1315	(73.4)	1401	(74.2)	1080	(79.5)
Nonurgent	2304	(19.2)	269	(15.6)	375	(20.9)	369	(19.8)	432	(26.8)	390	(21.8)	354	(18.7)	115	(8.5)
1	2668	(22.2)	328	(19.0)	356	(19.9)	378	(20.3)	361	(22.4)	449	(25.1)	533	(28.2)	263	(19.4)
2	3575	(29.7)	724	(41.9)	589	(32.8)	467	(25.1)	555	(34.5)	475	(26.5)	409	(21.7)	356	(26.2)
3	2914	(24.2)	438	(25.4)	429	(23.9)	492	(26.4)	265	(16.5)	401	(22.4)	472	(25.0)	417	(30.7)
4	2873	(23.9)	236	(13.7)	419	(23.4)	525	(28.2)	429	(26.6)	466	(26.0)	475	(25.1)	323	(23.8)
Comorbidities at decision																
Major conditions*‡*	2901	(24.1)	373	(21.6)	386	(21.5)	418	(22.4)	368	(22.9)	452	(25.2)	518	(27.4)	386	(28.4)
Other conditions*†*	2856	(23.7)	520	(30.1)	496	(27.7)	526	(28.2)	384	(23.9)	379	(21.2)	354	(18.7)	197	(14.5)
None	6273	(52.1)	833	(48.3)	911	(50.8)	918	(49.3)	858	(53.3)	960	(53.6)	1017	(53.8)	776	(57.1)
Coronary anatomy affected at decision																
Left main	1780	(14.8)	251	(14.5)	287	(16.0)	299	(16.1)	256	(15.9)	265	(14.8)	284	(15.0)	138	(10.2)
Multivessel*§*	8715	(72.4)	1361	(78.9)	1407	(78.5)	1418	(76.2)	1202	(74.7)	1320	(73.7)	1274	(67.4)	733	(53.9)
Limited∥	1535	(12.8)	114	(6.6)	99	(5.5)	145	(7.8)	152	(9.4)	206	(11.5)	331	(17.5)	488	(35.9)
Time between catheterization and decision for surgical revascularization (weeks)																
0–1	6651	(55.3)	726	(42.1)	932	(52.0)	1005	(54.0)	912	(56.6)	1093	(61.0)	1236	(65.4)	747	(55.0)
2–3	2066	(17.2)	422	(24.4)	377	(21.0)	350	(18.8)	269	(16.7)	245	(13.7)	215	(11.4)	188	(13.8)
4–5	1041	(8.7)	209	(12.1)	174	(9.7)	157	(8.4)	150	(9.3)	139	(7.8)	102	(5.4)	110	(8.1)
6–7	642	(5.3)	122	(7.1)	92	(5.1)	123	(6.6)	74	(4.6)	84	(4.7)	66	(3.5)	81	(6.0)
≥8	1630	(13.5)	247	(14.3)	218	(12.2)	227	(12.2)	205	(12.7)	230	(12.8)	270	(14.3)	233	(17.1)

^*^218 patients had unknown values for urgency at decision.

^‡^Congestive heart failure, diabetes mellitus, chronic obstructive pulmonary disease, rheumatoid arthritis, or cancer.

^†^Peripheral vascular disease, cerebrovascular disease, dementia, peptic ulcer disease, hemiplegia, renal disease, or liver disease.

^§^Two or three-vessel disease with stenosis of the proximal left anterior descending (PLAD) artery.

^∥^Two-vessel disease with no stenosis of the PLAD artery or one-vessel disease with stenosis of the PLAD artery.

For direct admissions, the trends in changes in the distributions of patient characteristics over periods were similar to wait-listed patients (Table 2). Later periods tended to have older patients (*p*<0.001), fewer urgent cases at decision (*p*<0.001), and more patients with major comorbidities at the time of decision (*p*<0.001). In contrast to wait-listed patients, later periods tended to have more direct admissions who had less limited coronary anatomy affected (*p*<0.001) and more decisions to operate within a week of catheterization (*p*<0.001). About two-thirds of direct admissions had a decision to operate within a week of catheterization across all calendar periods.

**Table 2:** Characteristics of 12,818 direct admissions, who had decision for coronary artery bypass grafting in British Columbia 1992–2005, by calendar period of decision

	**All periods**		**1992–1993**		**1994–1995**		**1996–1997**		**1998–1999**		**2000–2001**		**2002–2003**												**2004–2005**	
**Characteristic**	**(*n=*12818)**		**(*n=*1204)**		**(*n=*1474)**		**(*n=*1621)**		**(*n=*2089)**		**(*n=*2121)**		**(*n=*1940)**												**(*n=*2369)**	
Age group at decision (years)																
<50	920	(7.2)	111	(9.2)	97	(6.6)	129	(8.0)	142	(6.8)	157	(7.4)	139	(7.2)	145	(6.1)
50–59	2604	(20.3)	228	(18.9)	275	(18.7)	313	(19.3)	435	(20.8)	432	(20.4)	414	(21.3)	507	(21.4)
60–69	4354	(34.0)	442	(36.7)	569	(38.6)	571	(35.2)	715	(34.2)	676	(31.9)	615	(31.7)	766	(32.3)
70–79	4341	(33.9)	389	(32.3)	481	(32.6)	550	(33.9)	726	(34.8)	747	(35.2)	663	(34.2)	785	(33.1)
≥80	599	(4.7)	34	(2.8)	52	(3.5)	58	(3.6)	71	(3.4)	109	(5.1)	109	(5.6)	166	(7.0)
Sex*																
Men	10067	(78.5)	914	(75.9)	1104	(74.9)	1247	(76.9)	1659	(79.4)	1687	(79.5)	1525	(78.6)	1931	(81.5)
Women	2750	(21.5)	290	(24.1)	370	(25.1)	374	(23.1)	430	(20.6)	434	(20.5)	415	(21.4)	437	(18.4)
Urgency at decision																
Urgent	5944	(46.4)	727	(60.4)	944	(64.0)	900	(55.5)	1046	(50.1)	887	(41.8)	698	(36.0)	742	(31.3)
Semiurgent	6445	(50.3)	453	(37.6)	485	(32.9)	654	(40.3)	906	(43.4)	1148	(54.1)	1211	(62.4)	1588	(67.0)
Nonurgent	429	(3.3)	24	(2.0)	45	(3.1)	67	(4.1)	137	(6.6)	86	(4.1)	31	(1.6)	39	(1.6)
Institution at decision																
1	2437	(19.0)	89	(7.4)	206	(14.0)	295	(18.2)	381	(18.2)	473	(22.3)	427	(22.0)	566	(23.9)
2	2962	(23.1)	342	(28.4)	369	(25.0)	333	(20.5)	466	(22.3)	426	(20.1)	457	(23.6)	569	(24.0)
3	4964	(38.7)	417	(34.6)	623	(42.3)	838	(51.7)	870	(41.6)	799	(37.7)	683	(35.2)	734	(31.0)
4	2455	(19.2)	356	(29.6)	276	(18.7)	155	(9.6)	372	(17.8)	423	(19.9)	373	(19.2)	500	(21.1)
Comorbidities at decision																
Major conditions*‡*	5458	(42.6)	413	(34.3)	552	(37.4)	626	(38.6)	885	(42.4)	949	(44.7)	914	(47.1)	1119	(47.2)
Other conditions*†*	6248	(48.7)	708	(58.8)	809	(54.9)	885	(54.6)	1033	(49.4)	1027	(48.4)	857	(44.2)	929	(39.2)
None	1112	(8.7)	83	(6.9)	113	(7.7)	110	(6.8)	171	(8.2)	145	(6.8)	169	(8.7)	321	(13.6)
Coronary anatomy affected at decision																
Left main	3184	(24.8)	282	(23.4)	358	(24.3)	377	(23.3)	466	(22.3)	539	(25.4)	534	(27.5)	628	(26.5)
Multivessel*§*	8855	(69.1)	814	(67.6)	1019	(69.1)	1136	(70.1)	1501	(71.9)	1470	(69.3)	1307	(67.4)	1608	(67.9)
Limited∥	779	(6.1)	108	(9.0)	97	(6.6)	108	(6.7)	122	(5.8)	112	(5.3)	99	(5.1)	133	(5.6)
Time between catheterization and decision for surgical revascularization (weeks)																
0–1	8576	(66.9)	885	(73.5)	1021	(69.3)	991	(61.1)	1381	(66.1)	1465	(69.1)	1246	(64.2)	1587	(67.0)
2–3	2232	(17.4)	164	(13.6)	215	(14.6)	326	(20.1)	334	(16.0)	372	(17.5)	406	(20.9)	415	(17.5)
4–5	592	(4.6)	46	(3.8)	54	(3.7)	81	(5.0)	87	(4.2)	85	(4.0)	113	(5.8)	126	(5.3)
6–7	364	(2.8)	25	(2.1)	38	(2.6)	45	(2.8)	76	(3.6)	46	(2.2)	58	(3.0)	76	(3.2)
≥8	1054	(8.2)	84	(7.0)	146	(9.9)	178	(11.0)	211	(10.1)	153	(7.2)	117	(6.0)	165	(7.0)

^*^1 direct admission with decision date in 2004–2005 had unknown sex.

^‡^Congestive heart failure, diabetes mellitus, chronic obstructive pulmonary disease, rheumatoid arthritis, or cancer.

^†^Peripheral vascular disease, cerebrovascular disease, dementia, peptic ulcer disease, hemiplegia, renal disease, or liver disease.

^§^Two or three-vessel disease with stenosis of the proximal left anterior descending (PLAD) artery.

^∥^Two-vessel disease with no stenosis of the PLAD artery or one-vessel disease with stenosis of the PLAD artery.

When compared over calendar periods, wait-listed patients were more prevalent in the low risk group and directly-admitted patients were more prevalent in high risk group (see Table 3). The percentage of low risk patients accessing surgery through direct admission declined considerably in years when supplementary funding was provided.

**Table 3:** Prognostic risk of in-hospital death, by calendar period of surgery and type of access

**Calendar period of surgery**			**Risk ∗, %**				
**Access type**	**<1.0**		**1.0–3.0**		**>3.0**		***p* value*‡***
1992–1993							<0.001
Wait-listed	808	(58.4)	555	(40.1)	20	(1.4)	
Direct admission	559	(46.2)	581	(48.0)	70	(5.8)	
1994–1995							<0.001
Wait-listed	920	(61.3)	549	(36.6)	32	(2.1)	
Direct admission	694	(46.9)	673	(45.5)	112	(7.6)	
1996–1997							<0.001
Wait-listed	929	(58.2)	625	(39.2)	41	(2.6)	
Direct admission	761	(46.9)	776	(47.9)	84	(5.2)	
1998–1999							<0.001
Wait-listed	829	(52.9)	675	(43.1)	62	(4.0)	
Direct admission	867	(41.2)	984	(46.8)	251	(11.9)	
2000–2001							<0.001
Wait-listed	525	(37.6)	724	(51.8)	148	(10.6)	
Direct admission	413	(19.4)	1119	(52.6)	594	(27.9)	
2002–2003							<0.001
Wait-listed	691	(40.6)	876	(51.4)	136	(8.0)	
Direct admission	458	(23.6)	1037	(53.5)	444	(22.9)	
2004–2005							<0.001
Wait-listed	489	(37.6)	692	(53.2)	119	(9.2)	
Direct admission	461	(19.3)	1242	(52.1)	683	(28.6)	

^*^Based on Northern New England Cardiovascular Disease Study Group [22].

^‡^Compares the distribution of risk between wait-listed patients and direct admissions for each period.

### Access to surgery through wait-list registration

**Figure 1:**
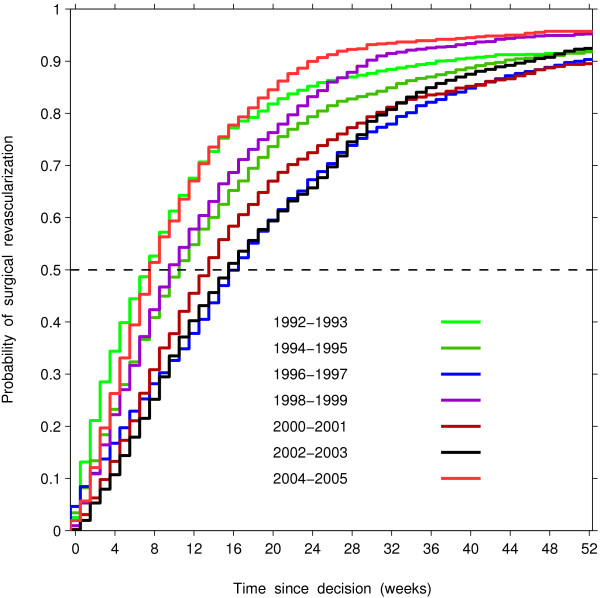
Estimated probability of surgical revascularization within a certain time of decision for surgical revascularization, by calendar period of decision, among patients who accessed surgery through registration on a wait list.

**Figure 2:**
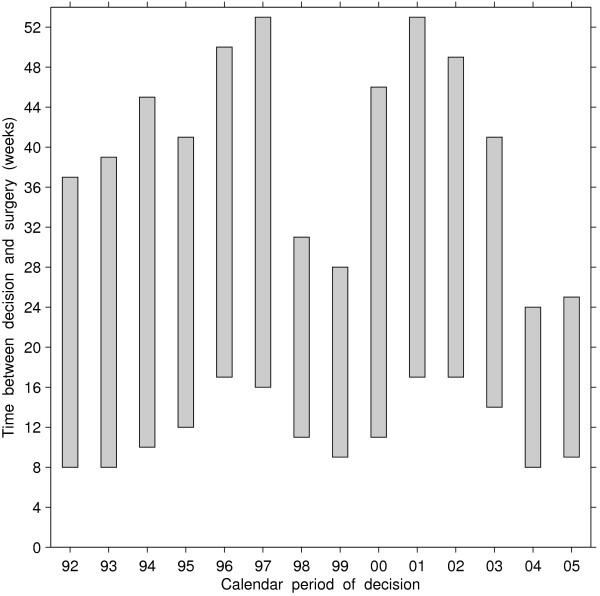
**Time between decision to operate and surgical revascularization among patients who accessed surgery through registration on a wait list, by calendar period of decision.** Bottom of bar = 50th percentile, top of bar = 90th percentile

Figure 1 shows the cumulative distribution functions of waiting time for each calendar period, which could be used to derive the number of weeks required for a specified proportion of patients to undergo the operation. The differences in the proportion of patients undergoing CABG within a certain time of decision were significant across periods (Log-rank statistic = 545.6, df=6, *p*<0.001), with longer waiting times when the decision was made in 1996–1997 and 2002–2003. The waiting times for these years were such that half of the wait-listed patients underwent surgery within 16 to 17 weeks, and 90% underwent surgery within 46 to 51 weeks. In contrast, during the other years about half of patients underwent surgery within 8 to 14 weeks of decision. Comparing the 1998, 1999, 2004 and 2005 cohorts (the periods when supplement funding was provided) with the rest, we observed a compression in access to surgery, i.e., reduction in the length of the waiting-time interval required for a specified proportion to undergo the operation. As measured by the difference between 90th and 50th percentiles of the wait time distributions, 40% of the 1998, 1999, 2004 and 2005 cohorts underwent surgery within 16 to 20 weeks following the median waiting time (50th percentile), while it took between 27 and 37 weeks for the cohorts in years when supplementary funding was not available (Figure 2).

**Figure 3:**
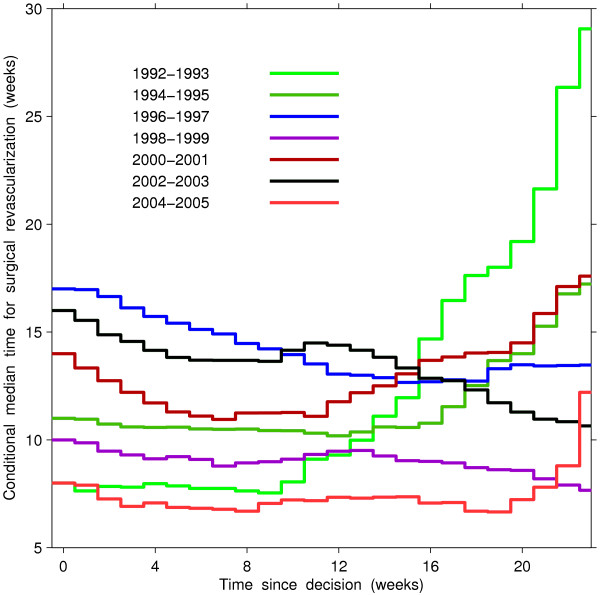
Conditional median time of surgical revascularization at a certain time since decision for surgical revascularization, by calendar period of decision, among patients who accessed surgery through registration on a wait list.

Periods 1992–1993 and 2004–2005 showed the shortest waiting time and showed a notable difference in changes of conditional remaining times between the periods (Figure 3). For each period, the remaining time during which half of the patients were expected to access surgery did not change for about the first 10 weeks since decision. After the first 10 weeks, the conditional median times for surgery remained relatively constant for period 2004–2005, but these times increased with longer waits for period 1992–1993. In contrast, in the two periods showing the longest waiting times, namely 1996–1997 and 2002–2003, the conditional median time decreased with the longer waits, indicating perhaps an active wait-list management in years when the supplementary funding was not provided.

Once wait-listed patients had a decision to operate, the average weekly number of operations was lowest in 1996–1997, 2000–2001, and 2002–2003 (Table 4). The average rate of undergoing surgery from a wait list was about 5 procedures per 100 patient-weeks in these periods with the lowest rates. After adjustment for patient-related factors, compared to the surgery rate in 1996–1997, the weekly odds that a patient would undergo operation were highest in 1992–1993, decreased to a low in 1996–1997, rose in 1998–1999, decreased to another low in 2000–2001 and 2002–2003, and then rose again in 2004–2005 (Table 4).

**Table 4:** Average weekly rate of coronary artery bypass procedure in relation to calendar period of decision to proceed with surgery, for patients registered on a wait list

**Type of access by**	**No. of**	**No. of**	**Total**	**Crude rate*‡***	**Crude OR*†***		**Adjusted OR *†§∥***	
**calendar period of decision**	**patients**	**procedures**	**waiting time*∗***	**(95% CI)**	**(95% CI)**		**(95% CI)**	
1992–1993	1726	1519	20462	7.4 (7.1–7.8)	1.7	(1.6–1.9)	2.0	(1.9–2.2)
1994–1995	1793	1545	26452	5.8 (5.5–6.1)	1.3	(1.2–1.4)	1.4	(1.3–1.5)
1996–1997	1862	1555	35054	4.4 (4.2–4.7)	1.0		1.0	
1998–1999	1610	1426	21266	6.7 (6.4–7.1)	1.5	(1.4–1.6)	1.5	(1.4–1.7)
2000–2001	1791	1509	31887	4.7 (4.5–5.0)	1.1	(1.0–1.2)	1.1	(1.0–1.2)
2002–2003	1889	1613	35447	4.6 (4.3–4.8)	1.0	(0.9–1.1)	1.0	(0.9–1.1)
2004–2005	1359	1172	14252	8.2 (7.8–8.7)	1.9	(1.7–2.0)	1.9	(1.8–2.1)
All periods	12030	10339	184820	5.6 (5.5–5.7)	–		–	

Abbreviations: OR = odds ratio; CI = confidence interval.

^*^To measure waiting times, patients were followed for a maximum of 52 weeks.

^‡^Per 100 patient weeks calculated as the number of procedures performed divided by the sum of waiting times.

^†^As measured by odds ratios derived from discrete-time survival models, adjusting for consecutive weeks of waiting (0.5–52 weeks).

^§^Adjusted for sex, age at decision, urgency at decision, institution at decision, comorbidities at decision, coronary anatomy at decision, and time between catheterization and decision.

^∥^218 patients with unknown values for urgency at decision were excluded.

### Access to surgery through direct admission

**Figure 4:**
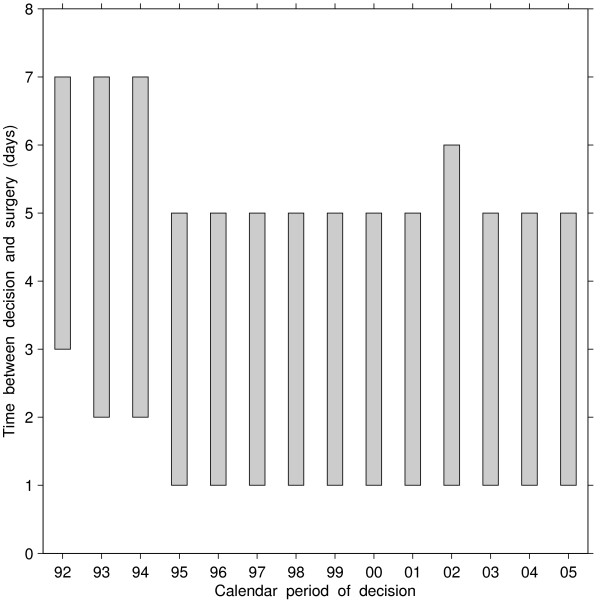
**Time between decision to operate and surgical revascularization among patients who accessed surgery through direct admission, by calendar period of decision.** Bottom of bar = 50th percentile, top of bar = 80th percentile

Among patients who had a decision to operate for CABG and were directly admitted to hospital, times between decision to operate and surgical revascularization were longest in the periods 1992–1994, but were much shorter than the times for wait-listed patients (Figure 4). During these periods 80% of patients underwent surgery within 7 days. Half of patients underwent surgery within 1 day of decision to operate and 80% within 5 days during periods 1995 and later.

Once direct admissions had a decision to operate, the average daily number of operations was lowest in 1992–1993 at about 23 procedures per 100 patient-days, rose to about 36 procedures per 100 patient-days in 1998–1999, after which the rate remained stable (Table 5). After adjustment for patient-related factors, compared to the surgery rate in 1996–1997, the daily odds of surgery were lower prior to 1996–1997, but higher after this period (Table 5).

**Table 5:** Average daily rate of coronary artery bypass procedure in relation to calendar period of decision to proceed with surgery, for direct admissions

**Type of access by**	**No. of**	**No. of**	**Total**	**Crude rate*‡***	**Crude OR*†***		**Adjusted OR*†§∥***	
**calendar period of decision**	**patients**	**procedures**	**waiting time*∗***	**(95% CI)**	**(95% CI)**		**(95% CI)**	
1992–1993	1204	1003	4228	23.7 (22.3–25.2)	0.7	(0.6–0.7)	0.7	(0.6–0.8)
1994–1995	1474	1270	4489	28.3 (26.7–29.8)	0.8	(0.7–0.9)	0.9	(0.8–0.9)
1996–1997	1621	1442	4173	34.6 (32.8–36.3)	1.0		1.0	
1998–1999	2089	1920	5301	36.2 (34.6–37.8)	1.1	(1.0–1.2)	1.2	(1.1–1.3)
2000–2001	2121	1895	5579	34.0 (32.4–35.5)	1.0	(0.9–1.1)	1.2	(1.1–1.3)
2002–2003	1940	1740	5145	33.8 (32.2–35.4)	1.0	(0.9–1.1)	1.1	(1.0–1.2)
2004–2005	2369	2140	6206	34.5 (33.0–35.9)	1.0	(1.0–1.1)	1.2	(1.1–1.3)
All periods	12818	11410	35121	32.5 (31.9–33.1)	–		–	

Abbreviations: OR = odds ratio; CI = confidence interval.

^*^To measure waiting times, patients were followed for a maximum of 7 days.

^‡^Per 100 patient days calculated as the number of procedures performed divided by the sum of waiting times.

^†^As measured by odds ratios derived from discrete-time survival models, adjusting for consecutive days of waiting (0–7 days).

^§^Adjusted for sex, age at decision, urgency at decision, institution at decision, comorbidities at decision, coronary anatomy at decision, and time between catheterization and decision.

^∥^1 patient with unknown value for sex was excluded.

### Time between catheterization and surgery

We found that for those who underwent CABG, times between catheterization and surgical revascularization were shorter among patients who accessed surgery through direct admission compared to access through wait-list registration. In the urgent group, half of direct admissions underwent surgery within 1 week, whereas half of wait-listed patients underwent surgery within 7 weeks. In addition, half of direct admissions and half of wait-listed patients underwent surgery within 1 and 13 weeks respectively, in the semiurgent group. In the nonurgent group these timeframes were 8 and 23 weeks, respectively. The weekly odds of surgery after catheterization were 4, 5, and 3 times higher among direct admissions compared to wait-listed patients in the urgent, semiurgent, and nonurgent groups, respectively, after adjustment for age, sex, hospital at catheterization, mode of admission at catheterization, comorbidity at surgery, hospital at surgery, and coronary anatomy at surgery. We also found that time between catheterization and decision to operate became shorter in the 2000s compared to the 1990s for the semiurgent group; the weekly rate of registration was 16% and 25% higher in 2000–2001 and 2002–2003 compared to 1996–1997. In the urgent group, these rates were 2.5 and 1.6 times higher in 2002–2003 and 2004–2005 compared to 1996–1997. The weekly rates of decision were not different across calendar periods in the nonurgent group, after adjustment.

## Discussion

Coronary revascularization is indicated to alleviate chest pain and to reduce the risk of death among patients who have limiting angina that persists despite optimal medical treatment and who have coronary anatomy that is suitable for the procedure. However, in healthcare systems that use wait lists to manage access to care, patients requiring non-emergency surgical revascularization may have to wait after the decision to operate. In this paper, we sought to determine whether the year of decision to proceed with non-emergency CABG had an effect on time to surgery in a health system with publicly-funded universal coverage for the procedure. We estimated temporal changes in the length of time that patients had to wait between decision to operate and the procedure itself over 14 years that included several years with increases in funding. We focused on isolated CABG surgery because access to surgery could have been managed differently for combined procedures than for isolated CABG. As a result, we did not consider data from 312 (2%) wait-listed patients and 211 (1%) directly-admitted patients whose procedure was not isolated CABG.

We found that during two periods when supplementary funding was available, 1998–1999 and 2004–2005, the weekly rate of undergoing surgery from a wait list was, respectively, 50% and 90% higher than in 1996–1997, the period with the longest waiting times. We also observed a reduction in the difference in 90th and 50th percentiles of the waiting-time distributions. Forty percent of patients in the 1998, 1999, 2004 and 2005 cohorts (years when supplementary funding was provided) underwent surgery within 16 to 20 weeks following the median waiting time, while it took between 27 and 37 weeks for the cohorts registered in the years when supplementary funding was not available. Among patients who were directly admitted to hospital, time between decision and surgery was longest in 1992–1993 and then steadily decreased through the late nineties. The rate of surgery among patients directly admitted to hospital was the highest in 1998–1999, and has not changed afterwards, even in years when supplementary funding was provided.

The most important contribution of this analysis is providing a more complete picture of access times for the patient population requiring surgical revascularization on a non-emergency basis in a health care system that budgets the number of CABG procedures and uses supplementary funding to reduce the number patients who have to wait for the procedure and their waiting times. We contrasted two pathways for accessing CABG. If angioplasty is not indicated when the cardiologist evaluates the arterial lesions on the coronary angiogram, then a cardiac surgeon is consulted to assess the patients’ suitability for CABG. Patients are transferred to an in-patient ward directly from the catheterization laboratory if expedited assessment is necessary and, if deemed suitable, these patients wait for the operation in hospital without registration on a wait list. Alternatively, a consultation with the surgeon is scheduled at a later date. Surgeons register on their wait lists patients who need CABG and for whom the operation can be safely delayed. To address this issue, we studied access to surgical coronary revascularization for non-emergency patients through direct admission to hospital at the surgeon’s discretion, and contrasted the total amount of time between cardiac catheterization and surgical revascularization for the two pathways: through a wait list and through direct admission.

In this analysis, a potential concern is the misclassification of the recorded urgency for treatment, because surgeons may manage access to surgery on the basis of various considerations, such as the best use of operating time or the availability of hospital resources. Therefore, the outcome might have been influenced by the individual surgeon’s threshold for accepting a patient for nonurgent treatment. It is plausible that the time to surgery may differ between patients treated by surgeons with a high volume of CABG procedures and surgeons who perform a diverse range of cardiac procedures.

We did not have access to detailed information about physicians’ decision-making on access to the procedure. To explore further the effect of the supplementary funding, we classified all patients using the algorithm developed by Northern New England Cardiovascular Disease Study Group [22]. The percentage of low risk patients accessing surgery through direct admission declined considerably in years when supplementary funding was provided.

More research is needed to evaluate whether waiting times for non-emergency surgery vary because of chance alone after adjustment for clinical factors and variation in supply. For example, it remains unclear whether directly admitting patients of low risk is done to circumvent long wait lists, or to substitute for cancellations on the operating room schedule.

Since 2002, percutaneous coronary intervention has become an increasingly common method of coronary revascularization, leading to a considerable change in the composition of patient population for both catheter-based and surgical procedures. We only had data for the period before 2005, and therefore our analysis could not adjust for changes in the proportional use of surgical revascularization over the past decade.

## Conclusions

Our study provides evidence that waiting times for non-emergency coronary bypass surgery shortened after supplementary funding was granted to increase volume of cardiac surgical care in a health system with publicly-funded universal coverage for the procedure. The effect of the supplementary funding was not uniform for patients that access the services through wait lists and through direct admissions. This might indicate that surgical services have used supplementary funding and direct admissions as two independent mechanisms to provide more treatments without delay. Considering that the hospitals had capacity to increase the number of operations, the supply-side initiatives indeed were effective in reducing waiting times. Perhaps it was an empirical way to find the level to budget the number of surgeries in the well defined population. In our view, some further options for improving access to cardiac care should include policies for effective management of patient flow.

## Competing interests

The authors declare that they have no competing interests.

## Author’s contributions

BS conceived the study concept and design, participated in analysis and interpretation, and drafted the manuscript. GF participated in data acquisition and critically revised the manuscript. LK participated in analysis and interpretation, and drafted the manuscript. AL participated in data acquisition. RS performed database analysis and has been involved in drafting the manuscript. BR performed statistical analysis and drafted the manuscript. All authors read and approved the final manuscript.

## Pre-publication history

The pre-publication history for this paper can be accessed here:

http://www.biomedcentral.com/1472-6963/12/311/prepub
